# Antimicrobial Nanomaterials: Why Evolution Matters

**DOI:** 10.3390/nano7100283

**Published:** 2017-09-21

**Authors:** Joseph L. Graves, Misty Thomas, Jude Akamu Ewunkem

**Affiliations:** 1 Department of Nanoengineering, Joint School of Nanoscience & Nanoengineering, North Carolina A&T State University and UNC Greensboro, Greensboro, NC 27401, USA; judeakamu@gmail.com; 2 Department of Biology, North Carolina A&T State University, Greensboro, NC 27411, USA; mthomas1@ncat.edu

**Keywords:** Antimicrobials, metals, acclimation, adaptation, evolution, genomics

## Abstract

Due to the widespread occurrence of multidrug resistant microbes there is increasing interest in the use of novel nanostructured materials as antimicrobials. Specifically, metallic nanoparticles such as silver, copper, and gold have been deployed due to the multiple impacts they have on bacterial physiology. From this, many have concluded that such nanomaterials represent steep obstacles against the evolution of resistance. However, we have already shown that this view is fallacious. For this reason, the significance of our initial experiments are beginning to be recognized in the antimicrobial effects of nanomaterials literature. This recognition is not yet fully understood and here we further explain why nanomaterials research requires a more nuanced understanding of core microbial evolution principles.

## 1. Introduction

There has been much interest in utilizing engineered nanomaterials for a variety of antimicrobial applications in agriculture and medicine. However, much of this work has been conducted by engineers and chemists who do not fully understand how biological systems respond to novel materials on either a physiological or an evolutionary time scale. For example, Soto-Quintero et al. (2017) stated:

Moreover, both hydrogel nanocomposite systems exhibited a more effective antibacterial activity against *P. aeruginosa* …than against *E. coli*…, as proven with the higher inhibition halo. The explanation of this fact could lie on the ability of *E. coli* to develop heavy metal resistance, particularly for silver.

[1]

To support this claim, they cited our 2015 paper, entitled Rapid evolution of silver nanoparticle resistance in *Escherichia coli* [2]. Unfortunately, this claim is not supported by the results we obtained and indicates that the authors have a fundamental misunderstanding of the underlying mechanisms of antimicrobial resistance. Citing this example is by no means an attempt to minimize the scientific accomplishments of these authors, but to draw attention to the fact that these kinds of errors are still commonplace in materials research when attempting to evaluate the efficacy of metallic nanomaterials against microbial growth [3]. This example allows us to make a broader point concerning how a more comprehensive understanding of the biology of microorganisms will result in the progression of materials science research to better fulfill its overall goals. Therefore, in this short essay we will attempt to clarify and provide the basic biology underlying antimicrobial resistance (specifically resistance to novel nanomaterials) and why this knowledge is crucial for understanding how to design antimicrobial materials that can have sustainable applications.

## 2. Physiological Acclimation and Evolutionary Adaptation

Homeostasis is a self-regulating core feature of biological organisms that allows them to, whenever possible, maintain their internal stability and keep a constant physiological state. There are a number of homeostatic process that must be preserved in order to guarantee the survival of the organism including iron homeostasis, metal homeostasis, pH homeostasis and membrane and lipid homeostasis [4,5,6,7,8]. Since the beginning of life on this planet, microbes have been exposed to metals—some necessary, but many that are toxic to the cells [9]. Iron is an example of a metal required for survival, but when found in sufficient quantities is toxic. In order to maintain homeostasis, bacteria have devised methods to control iron levels by upregulating/downregulating genes involved in a number of mechanisms. For example, in response to iron starvation, some secrete high affinity iron chelators to maximize uptake and alternatively, in response to toxic iron levels, some upregulate expression of iron detoxifying proteins and genes involved in efflux. The majority of this differential regulation is dependent on the ferric uptake regulator protein (Fur) [4]. For example, in *Escherichia coli* there are 7 iron acquisition systems that are controlled by 35 iron-repressed genes. These in turn are all regulated by Fur [4].

Virtually all bacteria have acquired genes that control their physiology, allowing them to resist toxic metal ions (Ag^+^, Cd^2+^, Hg^2+^, Ni^2+^ etc.). To deal with these toxic metals, many bacteria express metal-sensing transcriptional regulators that can sense both beneficial and toxic metals allowing them to adapt to their environments rather quickly and as a result, energy dependent efflux is the most commonly deployed metal resistance mechanism among microorganisms [5,9]. Our studies showed this for both ionic and nanosilver (Ag^+^) based resistance in *E. coli* [2,10]. Alternative to an increase in efflux, some other mechanisms of metal resistance include enzymatic transformations (oxidation, reduction, methylation, and demethylation) or expression of metal-binding proteins (metallothionein, SmtA, chaperone CopZ, SilE [9]). In addition, some clones may prevent toxic metal ions from entering their cells through downregulating expression of membrane transport proteins, of which we have found evidence to support [2,10,11,12].

Finally, bacteria may evolve persister phenotypes in which they slow their growth, or cease dividing in the presence of toxic materials to prevent the toxic consequences of the metals [13,14,15]. When this process is successful bacteria undergo physiological acclimation [16,17,18]. This can take place on the scale of minutes to hours and these changes are dependent not only on the species of bacteria but even on the specific strain. For example, a prior study measured gene expression changes in *E. coli* strain XL-1 blue exposed to silver nanoparticles embedded in zeolite membranes [19]. After 30 minutes of exposure they showed a 3.0–15.0 fold increases in the expression of 24 genes. As a cautionary tale of how physiological and evolutionary adaptation may not involve the same genes, while *E. coli* XL-1 blue and *E. coli* K12 MG1655 share many of their genes, none of those that were upregulated in that study were targets of selection in our work [2,10]. *E. coli* XL-1 blue is a cloning strain that has undergone significant genetic engineering in order to be optimized for cloning and molecular biology based experiments (Stratagene) in addition, their base phenotype is tetracycline resistant. This is in sharp contrast to *E. coli* K12 MG1655 which is a maintained laboratory strain with minimal mutation (PMID 9278503). It possible that these 25 gene changes observed in XL-1 blue might have been initial targets of selection in K12 MG1655 strain, but by generation 100 there was no evidence of any mutational changes being swept to higher frequencies in our populations. Alternatively, these are significantly different strains that will develop different strategies to resist heavy metal toxicity dependent on their base genetics.

Evolution by natural selection requires three elements: variation, heredity, and a struggle for existence. Since microbes maintain very large populations (10^8^–10^9^ per mL) there is always a great deal of genetic variation within their populations. The large population size guarantees that suitable numbers of mutations will always be present in microbial populations. A mutation, being a heritable change in the genetic information of an organism, most often occur due to a decreased fidelity in DNA polymerases, the enzymes responsible for DNA replication, making the process slightly prone to errors [20]. For example, the mutation rate in *E. coli* has been estimated at ~ 1 × 10^−10^ to 1 × 10^−9^ per genome per generation [21,22]. Therefore, the average *E. coli* cell with a genome size of 4.6 million base pairs, should be expected to display less than 1 mutation per cell. Most of these mutations will be neutral or have minimal effect for the cells in their environment [23,24]. The number of genetic variants we would expect in a population of 10^9^ microbes would be between 4.6 million on the high end to about 460,000 on the low end. This means that microbes usually contain ample amounts of mutational variation to respond to new environmental conditions.

Under all conditions in nature, bacteria are constantly engaged in a struggle for existence. If this were not the case, simply by geometric growth they would have exceeded the nutritional capacity of the earth a long time ago. Adding toxic metals to the microbial environment produces a struggle for existence directly related to the nature of the toxin. The organisms that cannot prevent metal entry, respond with efflux, or detoxify internally are killed or rendered infertile. The initial response to toxins is always physiological, however there is always genetic variation among the microbes that manage to survive in the presence of the toxin and some of these genetic variants will show greater reproductive success under these conditions. Any resistance mechanism that is encoded in the genome is passed on to the next generation (heritable, vertical gene transfer).

It may also be spread to unrelated bacterial clones within a species or to other species of bacteria through horizontal gene transfer if the toxin remains in the environment. As a result, the variants best capable of reproduction (differential reproductive success) will rapidly dominate the population through generational time until the entire population carries these specific genetic variants. This process is called natural selection and it is the sole driving force of evolutionary adaptation and due to the size of microbial populations, the course of evolution in these organisms is exceedingly rapid [25].

## 3. Evolution Is Always Occurring

The fact that evolution is always occurring in bacterial populations means that researchers who wish to understand how a given nanomaterial is going to impact microbes must take this into account in their experimental design and thus far, this is a weakness in the material scientists field [3]. For example, Brown et al. utilized silver nanoparticles (AgNP) functionalized with ampicillin (AMP) to reduce multidrug resistant populations of *Enterobacter aerogenes* and *Staphyloccocus aureus* [26]. They were able to show a reduction of the AgNP-AMP treated bacterial strains to < 1 colony forming unit (CFU) with increasing concentration (4.0 and 20.0 μg/mL) of AgNP-AMP in their samples compared to >8 CFU in controls not treated with AgNP-AMP after 8 hours. On the face of it, these results are promising, however, the authors did not discuss the possibility that bacterial strains could evolve resistance to the combination treatment of AgNP-AMP. This possibility is evident in the data they reported. For example, at concentrations of 2.0 and 1.0 μg/mL the strains of *Vibrio cholera*, *E. aerogenes*, and *S. aureus* (MRSA) showed only slight reduction relative to the controls (at 1.0 μg/mL, 9.19 ± 0.4, 7.48 ± 0.08, 8.67 ± 0.05 compared to controls 9.23 ± 0.04, 9.56 ± 0.01, 9.06 ± 0.13 respectively and at 2.0 μg/mL for *E. aerogenes* and *S. aureus* (MRSA) 5.36 ± 0.06, 4.64 ± 0.14 compared to controls 9.56 ± 0.01, 9.06 ± 0.13 respectively). It is precisely when bacteria experience toxin conditions that are sub minimal inhibitory concentration (MIC) that the possibility of the evolution of resistance is highest and there is evidence that this occurs widely in nature. For example, one study found that bacteria (*Klebsiella plantacola*) isolated from the Kizilirmak river in Turkey displayed resistance to 15 antibiotics (ampicillin, amoxicillin/clavulanic acid, aztronam, erythromycin, imipenem, oxacillin, pefloxacin, penicillin, piperacillin, piperacillin/tazobactam. rifampicin, sulbactam/cefoperazone, ticarsillin, ticarsillin/clavulanic acid, vancomycin) and 11 heavy metals (aluminum, barium, copper, iron, lead, lithium, manganese, nickel, silver, strontium, and tin) [27]. This resistance phenotype originates from exposure to sub-MIC concentrations of both the antibiotics and metals entering the waste stream and then the river. Dobias and Bernier-Latmani 2013 showed that silver nanoparticles could continue to release ionic silver into natural waters for ~4 months [28].

Shared antibiotic and metal resistance can evolve via two mechanisms, either the acquisition of a plasmid carrying genes for both such as members of the IncHI-2 incompatibility group or de novo pleiotropic mutations that impact both traits [29]. We have recently shown an example of the latter possibility in our research laboratory [30]. Utilizing experimental evolution to increase Fe^2+^ and Fe^3+^ resistance in a naïve strain of *E. coli* K12 MG1655. We have demonstrated that our Fe^2+^-resistant strains are also resistant to ampicillin, polymyxin B, and rifampicin relative to controls and the Fe^3+^-resistant strains showed greater resistance to chloramphenicol, polymyxin B, and rifampicin relative controls. We have conducted whole genome sequencing in these strains and have found some selective sweeps in the Fe^2+^-resistant and Fe^3+^-resistant lines that could account for this pleiotropy. Specifically, we have found mutations in *dnaK* (helps to deal with osmotic shock from reactive oxygen species damage, *murC* involved in cell wall synthesis, and *mrdA* catalyzes cross-linking of the peptidoglycan cell wall) and *tolC,* which is involved in responses to antibiotics and ion transmembrane support, that could account for antibiotic resistance [31].

## 4. Genomes Matter

A consistent feature of materials science research is the “off-the-shelf” approach to utilizing biological materials. For example, while Nagy et al. (2011) was an excellent mechanistic study, the authors spent a great deal of time describing the synthesis of their AgNPs in zeolite, characterization of AgNP-ZM, and how they conducted the DNA expression and gene expression microarrays [19]. They did not explain why they chose *E. coli* XL-1 blue or what its genomic characteristics were relative to what they wanted to study. In addition, the fact that it carries a tetracycline resistant plasmid could significantly alter the results that they would have seen in a wild type strain considering the pleiotropy that we have observed in our current studies. In contrast, our studies utilized *E. coli* K12 MG1655, a maintained laboratory strain with minimal mutations, because this strain contained only rudimentary silver resistance, encoded by the *cusCFBARS* gene cluster which is found in all *E. coli* strains [2,10,32].

In Soto-Quintero et al. (2017) the authors utilized specific strains of *Pseudomonas aeruginosa* (ATCC 25922) and *Escherichia coli* (ATCC 2785) [1]. However, it is possible that there was a typological error, as neither strain is present in Entrez PubMed nor in the ATCC database. *Escherichia coli* strain (ATCC 25922) is present in the ATCC database and this strain can be found in the Entrez Pubmed nucleotide database, as opposed to the fact that there is no entry for *Pseudomonas aeruginosa* (ATCC 25922) found on the ATCC site or the Entrez Pubmed Database. Despite the possible error in strain designation, it is more problematic that the materials and methods section of this paper did not fully describe the culture conditions under which these bacteria were grown. In these experiments, it is necessary for the bacterial strains to, in the short run display physiological acclimation, or in the long run adapt not only to the experimental conditions but to all of the environmental conditions in which the researcher is using, and therefore the details of the experiments are essential for understanding the context of the results. For example, it is important to report culture temperature, rpm in the shaking incubator and whether it was carried out in a shaker incubator. Furthermore, it is important to discuss silver resistance mechanisms in subject species, particularly these specific strains. This is crucial as horizontal gene transfer can lead to great genomic differences between the strains within a bacterial species, which is why modern microbiology more properly views bacterial species as being composed of pangenomes. For example, the pangenome of *Pseudomonas aeruginosa* can be between 6.5 × 10^6^–7.4 × 10^6^ base pairs and there are 16,820 non-redundant genes, of which 2503 are consider part of the core genome [33]. In additional 9108 genes are considered part of its accessories genome. The average number of genes that are unique per strain are about 16. A cursory examination of the *Pseudomonas aeruginosa* core genome reveals that it has the EnvZ-OmpR pathway which was shown to contribute to silver resistance [2,10,33]. In addition, this bacterium has a copper efflux pump system (Cu^2+^) encoded by the *copRS* and *copCBA* system [34]. Mutations in this system could easily repurpose the pump and lend itself to silver efflux (copper and silver are in the same column of the periodic table).

The fact that bacteria species and strains are not simply one thing nor identical, directs us to the fact that the pangenome has a profound effect on how we interpret the results of any antimicrobial treatment. Indicating that for any specific bacterium we can expect a set of specific results for a particular antimicrobial (silver, iron, traditional antibiotic, etc.) and we cannot necessarily generalize that result to how we would expect that antimicrobial to work against another bacterial strain within the pangenome. For example, the bacterium *Cupriavidus metallidurans* is actually specialized for metal resistance and specifically the CH34 strain carries over 20 different metal ion resistances [35]. These metal resistance genes can be found on the chromosome, on plasmids and on mobile transposable elements which therefore gives it the opportunity to transfer these genes to non-resistant strains. In addition to CH34, there are many other strains and biotypes of *Cupriavidus metallidurans* that have been isolated and their resistance profile can change significantly dependent on the metal rich environment from which they were isolated [36]. Thus, all antimicrobial research should pay careful attention to which strains are being used and to accurately describe the components of the genome under observation. There was no reason to suppose that the differences observed in Soto-Quintero et al. (2017) resulted from the greater capacity of *E. coli* to “develop” anti-silver resistance compared to *P. aeruginosa*. This conclusion is at best overstated, and at worst incorrect, for several reasons including the need to precisely describe the phenomenon under observation.

If an antimicrobial material is being tested within one generation, you are observing physiological acclimation or the failure of such a phenomenon. If an antimicrobial material is being evaluated over multiple generations then you are observing evolutionary adaptation, or the failure of the phenomenon. In either case, what you are observing is intimately tied to the choice of bacterial strains used. In the case of *P. aeruginosa*, there are at least 181 strains within its pangenome, *E. coli* may have >60 strains and these strains have been evolving separately for a very long time [37]. For example, *E. coli* and *Salmonella enterica* are close relatives whose last common ancestor lived ~100 million years before the present day, whereas *E. coli K12* and *E. coli O157:H7* shared a common ancestor ~4.6 million years ago [37]. To give some context, this is longer in chronological time than the last common ancestor of *Australopithecus africanus* and the hominids (*habilis*, *ergaster*, *sapiens*) [22]. In fact, this is much longer in evolutionary time, as evolution occurs by generations not chronological years. *E. coli* can typically grow 6 generations in a day, whereas human generations are ~15 years. Finally underscoring how different bacterial strains can be within the same species is the fact that bacteria can receive genes by horizontal transfer from even distantly related bacteria (phage transfection, plasmids, transformation) [38,39]. This means that we must be cognizant of the genomic composition of the bacteria we are testing with nanomaterials. One size certainly does not fit all, and even if it did initially, microbial evolution insures that this will not remain the case.

## 5. Conclusions: How Can We Develop Sustainable Nano-Antimicrobials?

The point of this discussion has been to outline the crucial processes that are always operating in the microbial world. Much of the material above has focused on bacteria (prokaryotes) but apply with equal force with regards to applications designed to control other single celled organisms, such as apicomplexans, microsporidians, amoebas, or sporozoan parasites [40]. Despite our anthropocentric bias, life on this planet has always been primarily microbial and these microbes exist within virtually every habitat of the biosphere. In addition, these microorganisms have consistently evolved resistances to virtually every naturally employed biocide, such as those originating from soil dwelling bacteria such as *Actinomycetes* and to those developed by humans, indicating that antibiotic resistance was widespread in nature long before humans began to deploy them, this also true of metal resistance [9,39,41].

If this is true, then how exactly can we design sustainable antimicrobial treatments using nanomaterials? Certainly, there is great interest in this scientific enterprise. A recent review summarizes attempts at employing various nanomaterials (ZnO, Ag, Cu, Fe_3_O_4_, Al_2_O_3_, TiO_2_, SiO_2_, and chitosan) as antimicrobials [42]. Table 1 cites other examples of nanomaterial applications that are not cited in that review. Evidence has shown that single substance approaches are generally doomed to failure and that relatively simple genomic changes were required to confer resistance to compounds such as ionic or nanosilver [2,10,43]. Nagy et al. (2011) illustrated the correct approach to retarding the spread of resistance through combinational approaches, e.g., using silver and an antibiotic [19]. This study did not however consider how resistance to this combination approach might proceed. For example, this can occur by simply the combined probability of a clone acquiring a mutation against silver and the specific antibiotic used. The probability of this occurring de-novo is very low, but with large population size in bacterial populations this is entirely possible. In addition, there are plasmids that carry both metal and antibiotic resistance genes, so the probability of a clone acquiring both resistances at once is greatly increased. In the case of HIV therapy, the combination antiviral drug approach has been used since 1995 and has successfully reduced but not completely eliminated resistant strains of HIV [44]. Others have also shown the importance of combinational approaches in designing drugs that specifically target and shut down a variety of processes in bacteria in order to increase their susceptibility to currently available antimicrobial therapies [45,46].

The value of experimental evolution approaches is that they demonstrate potential targets that could be utilized to retard the spread of resistance. We currently have work that shows that bacteria that have resistance to silver, are at a great disadvantage when evolving resistance to excess Fe^2+^ and Fe^3+^ ions [30]. The results were counter intuitive in that the mechanisms associated with silver and iron toxicity are highly similar in *E. coli* (Table 2). This would suggest that a combination approach of using iron, an essential micronutrient, and silver, which is always toxic, could significantly retard the rate at which resistance evolves. This result is also supported by the theory of how antimicrobials can be employed in combination and suggests that models of antimicrobial sustainability must consider whether the combinations are synergistic, additive, or antagonistic [47]. Contrary to what one might expect, extinction rates are predicted to be greatest when the antimicrobial combination is antagonistic as synergistic or additive combinations may be more effectively resisted by common physiological responses which can be engendered by genes with pleiotropic effects (as we suspect we are seeing in our Fe^2+^/Fe^3+^-selected lines relative to antibiotics). However, antagonistic effects might engender a genomic tug of war in which the clones will not be able to serve both masters. It is here where nanomaterials may offer great opportunities for the development of sustainable approaches, especially if nanomaterials researchers are cognizant of the complexity of microbial responses to toxic materials. It is our contention that combination approaches that utilize both the diversity of elements (silver, copper, iron, gold, fullerenes, etc.), and biologics (bacteriophage and antibiotic compounds), as well as shape (nanoplates, nanodarts, nanospikes) offer the best opportunity to engineer more sustainable antimicrobial treatments.

## Figures and Tables

**Table 1:** Summary of select studies of the antimicrobial effects of nanomaterials.

Chemistry	Organism	Mechanism	Reference
Nano-Al_2_O_3_	*E. coli*, *Salmonella* spp.	Plasmid transfer	Qiu et al., 2012 [48].
Ag-montmorillonite	Fruit salad microbiome	Prolongs shelf life	Costa et al., 2011 [49].
AgNP-sulfidation	*E. coli*	Reduces growth inhibition.	Reinsch et al., 2012 [50].
AgNP-antibiotics	Enterobacteriaceae	Restores antibiotic activity	Panáček et al., 2016 [51].
AgNP, AuNP	*E. coli*, *bacillus* Calmette-Guérin	Growth reduction	Zhou et al., 2012 [52].
AgNP, CuNP	*E. coli*, *B. subtilis*, *Staphylococcus aureus*	Growth reduction	Ruparelia et al., 2007 [53].
AgNP, ZnONP	*E. coli*, MS2 bacteriophage	Growth reduction	You, Zhang, and Hu 2011 [54].
Binary Ag/CU NP	Bacteria and fungi	Growth reduction	Eremenko et al., 2016 [55].
Ag Carbene complexes	*Acinetobacter baumanii*, *P. aeruginosa*, *S. aureus*, *Bulkholderia cepacia*, *Klebsiella pneumoniae*	Bactericidal	Leid et al., 2011 [56].
CeO_2_, TiO_2_, Ag, Au, NPs	Wastewater microbiome	Growth, metabolism reduction	Garcia et al., 2012 [57].
γ-Fe_2_O_3_ NPs	*E. coli*	Bactericidal, Genomic impact	He et al., 2011 [58].
α-Fe_2_O_3_ NPs	*S. aureus*, *E. coli*, *P. aeruginosa*, *Serratia marcescens*	Bactericidal	Ismail et al., 2015 [59].
fullerenes	*E. coli*	Respiratory activity	Chae et al., 2009 [60].
Au NPs	*Coelastrella sp.*, *Phormidium sp.*	Bioaccumulation	MubarekAli et al., 2013 [61].
ZnO NPs and microwaves	*Brassica chinensis* microbiome	Sterilization	Liu et al., 2014 [62].

**Table 2:** Mechanisms of silver and iron toxicity in bacteria.

Mechanism	Ag	Fe
Reactive oxygen species	+	+
Binding to thiol groups	+	?
Transcription/Translation	+	+
Cell wall/membrane damage	+	+
Interfering with respiration	+	+
Release of cellular components	+	+
